# Effectiveness of a Reduced-Risk Insecticide Based Bed Bug Management Program in Low-Income Housing 

**DOI:** 10.3390/insects4040731

**Published:** 2013-11-28

**Authors:** Narinderpal Singh, Changlu Wang, Richard Cooper

**Affiliations:** Department of Entomology, Rutgers University, New Brunswick, NJ 08901, USA; E-Mails: nsingh@aesop.rutgers.edu (N.S.); rcooper@aesop.rutgers.edu (R.C.)

**Keywords:** bed bug, low-income housing, reduced-risk, monitoring

## Abstract

Bed bug (*Cimex lectularius* L.) infestations are becoming increasingly common in low-income communities. Once they are introduced, elimination is very difficult. As part of the efforts to develop effective and safe bed bug management programs, we conducted a laboratory study evaluating the efficacy of a reduced-risk insecticide—Alpine aerosol (0.5% dinotefuran). We then conducted a field evaluation of a reduced-risk insecticide based integrated pest management (IPM) program in low-income family apartments with young children. In laboratory evaluations, direct spray and 5 min exposure to dry Alpine aerosol residue caused 100.0 ± 0.0 and 91.7 ± 8.3% mortality to bed bug nymphs, respectively. Direct Alpine aerosol spray killed 91.3 ± 4.3% of the eggs. The IPM program included education, steam, bagging infested linens, placing intercepting devices under furniture legs and corners of rooms, applying Alpine aerosol and Alpine dust (0.25% dinotefuran, 95% diatomaceous earth dust), and regularly scheduled monitoring and re-treatment. Nine apartments ranging from 1–1,428 (median: 29) bed bugs based on visual inspection and Climbup interceptor counts were included. Over a 6-month period, an average 172 g insecticide (Alpine aerosol + Alpine dust) was used in each apartment, a 96% reduction in pesticide usage compared to chemical only treatment reported in a similar environment. The IPM program resulted in an average of 96.8 ± 2.2% reduction in the number of bed bugs. However, elimination of bed bugs was only achieved in three lightly infested apartments (<30 bed bugs at the beginning). Elimination success was closely correlated with the level of bed bug populations.

## 1. Introduction

Bed bugs, *Cimex lectularius* L. (Hemiptera: Cimicidae), are a significant public health pest in the U.S. and are very difficult to control [1]. Nationwide, low-income housing communities are experiencing higher number of bed bug problems than the rest of the society [2]. Once bed bugs are introduced, elimination is very difficult and often requires numerous pesticide treatments, non-chemical treatments, disposal of furniture and other personal belongings [3,4]. Residents’ lack of education and awareness to identify and report infestations early makes the bed bug control more difficult and expensive [4]. Potter *et al.* [5] reported 30% of the people surveyed did not react to bed bug bites while bed bugs were present. Unnoticed infestations go untreated and develop into larger infestations and spread within [4] and among buildings. Heavy infestations are more difficult to control than light infestations [6,7]. These chronic bed bug infestations in low-income communities can endanger the financial stability of the property, health of the residents, and further serve as the source of new infestations in our society.

Based upon our experience, most affordable housing management teams in New Jersey use low-bid and low quality pest control services that only suppress bed bug infestation levels, but do not necessarily reduce or eliminate the infestations, which in turn promote population rebound and spread. A recent study shows pest control contractor’s monthly service was significantly less effective than integrated pest management (IPM) treatment provided by researchers [8]. Low-income residents often do not know the options available to eliminate bed bug infestations successfully. Most people and professionals often rely on pesticides particularly pyrethroids to control bed bugs [4,6]. Pyrethroids, alone, are not very effective in eradicating bed bugs due to insecticide resistance among bed bug populations [9,10,11,12]. Moreover, frequent use of insecticides in sleeping and resting areas poses high risk of human pesticide exposure. Thus selection of a reduced-risk and effective insecticide is crucial for successful management of bed bugs without adversely affecting the occupants’ health from insecticide applications.

Non-chemical tools/methods such as steam, mattress encasement, de-cluttering, discarding severely infested items, and frequent laundering are effective elements in bed bug management [3,13,14]. Chemicals used for bed bug control include various insecticide sprays and dusts [15,16]. None of these methods alone can effectively eliminate bed bugs. An IPM approach combining education, prevention, non-chemical, and chemical methods is recognized as the most effective approach for managing bed bug infestations [3,6]. Wang *et al.* [3,7,8] demonstrated the effectiveness of non-chemical and chemical treatment based IPM programs in one-bedroom apartments occupied by elderly tenants. At present, there is no report on low-risk and effective IPM programs for bed bug elimination in family-style low-income housing with multiple bedrooms and multiple occupants. 

In an effort to design and implement a reduced-risk pesticide based IPM program for low-income housing, we evaluated the efficacy of a reduced-risk pesticide (Alpine aerosol, a.i. 0.5% dinotefuran, Whitmire Micro-Gen Research Laboratories, St. Louis, MO, USA) against bed bugs in the laboratory. Then we implemented a reduced-risk insecticide based IPM program in low-income family-style housing where most apartments had children present. 

## 2. Experimental Section

### 2.1. Insects

A bed bug strain (Indy) was collected from infested apartments in Indiana in 2008. Bugs were maintained in plastic containers (5 cm diameter and 4.7 cm height) with folded paper as harborages at 26 ± 1 °C, 40 ± 10% relative humidity (RH), a 12:12 h (L:D) photoperiod, and fed weekly on defibrinated rabbit blood using a Hemotek membrane-feeding system (Hemotek Ltd., Accrington, UK). Bugs were deprived of blood for one wk prior to bioassays. 

### 2.2. Efficacy of Alpine Aerosol in Laboratory Assays

#### 2.2.1. Direct Spray against Bed Bug Nymphs

**Figure 1:**
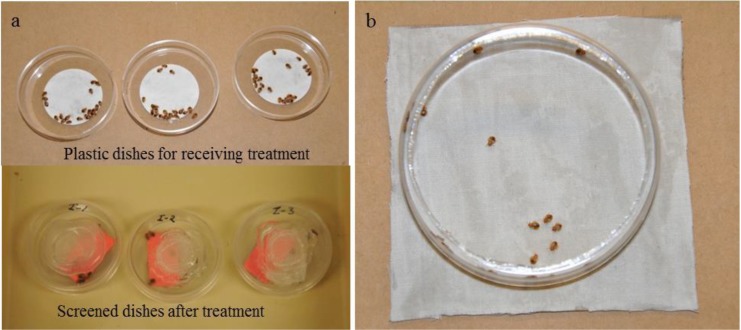
Experimental set up for evaluating the efficacy of Alpine aerosol: (a) Direct spray bioassay. (b) Dry residue exposure bioassay.

Twenty Indy strain bed bug nymphs (4th–5th instars) were placed on filter paper in a small plastic dish (5.5 cm diameter and 1.5 cm height) (Figure 1a). They were sprayed with Alpine aerosol at approximately 4.07 mg/cm^2^ (1 gallon/1,000 ft^2^). Bed bugs in the control group were sprayed with water at 4.07 mg/cm^2 ^using a Mainstays mister spray bottle (Walmart, Bentonville, AR, USA). All 20 bugs were immediately transferred to clean 1.5 cm diameter screened plastic petri dishes with a paper harborage after treatment (Figure 1a). The petri dishes were held in a room at 26 °C with 40%–50% RH, and a photoperiod of 12:12 h (L:D). Each treatment was replicated three times. Mortality data were taken at 1, 3, 5, 7, 10, and 14 d after treatment. A bed bug was considered dead if there was no movement when prodded with forceps.

#### 2.2.2. Direct Spray against Bed Bug Eggs

Two or three days old Indy strain eggs were taken out from rearing containers. In each replication, 22–26 eggs along with the paper substrate were sprayed following the same procedure as described in Experiment 2.2.1. Each treatment was replicated three times. Egg hatching and mortality of the nymphs emerged from eggs were recorded at 5, 7, 10, and 14 d after treatment.

#### 2.2.3. Dry Residue Exposure against Bed Bug Nymphs

Alpine aerosol was applied to 10 cm by 10 cm cardboard panels covered with white cotton fabric at approximately 4.07 mg/cm^2^. The control panels were sprayed with water using a spray bottle at approximately 4.07 mg/cm^2^. After 1 d, bed bugs were released onto the treated fabric and confined with a plastic ring (9 cm diameter and 2 cm height) for 5 min (Figure 1b). The bugs were then transferred to clean petri dishes following the procedures described in Experiment 2.2.1. Twenty 4th–5th instar nymphs were used in each replication. Each treatment was replicated three times. Mortality was recorded at 1, 3, 5, 7, 10, and 14 d after exposure.

### 2.3. Effectiveness of a Reduced-Risk Insecticide Based Bed Bug Management Program in Low-Income Housing

The field study was conducted in an apartment complex located in New Brunswick, NJ between June and December 2012. The two-story high buildings had 252 units occupied mostly by low-income families. Each apartment had 1-3 bedrooms. One-bedroom apartments were occupied by seniors, while two to three-bedroom apartments were occupied by families with young children. Prior to this study, bed bug infestations were treated by a pest control contractor based on complaints received from residents. Only three units in our study received bed bug treatment from the contractor within the previous 12 months. A few apartments had bed bugs for more than one year at the time we started the project. A total of 18 apartments were included in this study. They either had past or current infestations at the time of the study based on housing staff’s records and resident reports. Climbup^®^ Insect Interceptors (Susan McKnight, Inc., Memphis, TN, USA), referred to hereafter as “interceptors” were installed under the legs of beds and upholstered furniture, as well as in bedroom closets, the bathroom, and the corners of bedrooms and the living room in each apartment and inspected after 2 wk to determine the initial bed bug counts. Interceptors are an effective tool for detecting low level bed bug infestations and for evaluating the effectiveness of bed bug management programs [3,4]. An interview with residents was conducted at the initial installation of monitors. The residents were asked about the infestation history, symptoms from bed bug bites, self-control methods, if they had received professional pest control service, and their level of satisfaction with previous pest control service. A three page brochure (both English and Spanish) was provided to all residents. The brochure included: (a) bed bug identification, symptoms of bed bug bites and their impact on human health; (b) bed bug biology and behavior, typical signs of bed bug infestation; (c) how to prevent introducing new bed bugs; (d) how to control existing infestations by both chemical method and non-chemical (*i.e*., laundering, de-cluttering *etc.*) methods. Extra copies of the brochure were provided to the front office for residents to take free of charge. Residents were asked to follow the recommendations in the brochure to help prevent new introductions of bed bugs and to help eliminate existing infestations. 

Nine apartments (1 one-bedroom, 2 two-bedroom, 6 three-bedroom) were detected with bed bugs using a combination of interceptors and visual inspection. Each of the nine apartments was monitored for bed bug activity by installing an average of 24 ± 2 interceptors. Most of the interceptors (18.0 ± 2.6) were installed under the legs of beds and upholstered furniture. A small number of interceptors (6.0 ± 1.1) were placed in corners of the living rooms and bedrooms, bedroom closets, and the bathroom to aid in detection. An average of four persons were living in each of the eight units. The 9th unit was a one-bedroom apartment occupied by an elderly person. Among these, five units had young children, and four units had cats. Seven out of nine residents tried to control bed bug infestations by using pyrethroid, alcohol, essential oil sprays or insect foggers. Two residents discarded furniture in the past. We asked the residents to stop using any pesticides. Within 2 wk after the initial survey, the apartments were treated using the following: steam (The Steamax, Amerivap Systems, Dawsonville, GA, USA), hand removal during visual inspection, bagging infested bed sheets and linens, Alpine aerosol, and Alpine dust (0.25% dinotefuran, 95% diatomaceous earth dust, Whitmire Micro-Gen Research Laboratories, St. Louis, MO, USA). The IPM protocol also called for installation of mattress and box spring encasements on all beds, to be supplied by property management. Approximately one month into the study, property management provided encasements for only 6 of the 27 beds in the nine apartments. The six encasements were installed at the time they were made available. Following the initial treatment, apartments were placed on a biweekly inspection schedule. Due to obstacles associated with access to the apartments, in some instances up to one month’s time passed between follow-up visits. During each follow-up visit, the apartment was visually inspected for bed bugs and the interceptors were examined. Interceptors were wiped clean with cotton balls dabbed with talcum powder or replaced as necessary. Additional Alpine dust, Alpine aerosol, or steam was applied when >10 bed bugs were found during follow-up inspections. The Alpine aerosol was only applied to cracks on furniture, box springs, and sofa seams where live bed bugs were found. Alpine dust was applied to the perimeter of bedrooms and living rooms, electrical outlets, and cracks or crevices in walls. The six apartments adjoining the four heavily bed bug infested apartments were also inspected biweekly for one month using interceptors and visual inspections.

During each visit, infested bed sheets and linens were bagged and residents were asked to launder the bagged items. Residents were also asked to assist our control efforts through weekly laundering of bed linens and by reducing clutter. Three criteria were set to determine resident cooperation levels: (a) weekly laundering of bed linens, (b) reducing clutter, and (c) sanitation. Criteria a and b are considered by bed bug experts as essential measures. Criteria c is less critical but general sanitation can have an impact on pesticide efficacy. Cooperation levels were ranked into three categories: (1) very cooperative: residents meeting all three criteria; (2) somewhat cooperative: residents meeting one or two criteria; (3) not cooperative: residents not meeting any of the criteria. The technician time (time in an apartment multiplied by the number of technicians) spent servicing each apartment was recorded. The follow-up inspections ended in December 2012, about 6.5 months after initial treatment of the first batch of enrolled apartments. One apartment was initially treated in September and one apartment was initially treated in November. These two apartments received fewer follow-up visits than the other apartments and were still infested at the end of the program. Shortly after bed bugs were no longer found in an apartment for 2–3 consecutive visits or at the end of the program, we interviewed the residents about their bed bug knowledge, if they were still being bitten by bed bugs, satisfaction to our program, and any suggestions that they had. 

### 2.4. Statistical Analysis

For laboratory assays, the Abbott [17] formula was used to calculate corrected mortality: Corrected mortality = 
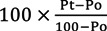
, where *Pt* = mortality in the treatment; *Po* = mortality in the control. Mean and standard error values for corrected mortality and bed bug counts were calculated [18]. 

## 3. Results and Discussion

### 3.1. Efficacy of Alpine Aerosol in Laboratory Assays

**Figure 2:**
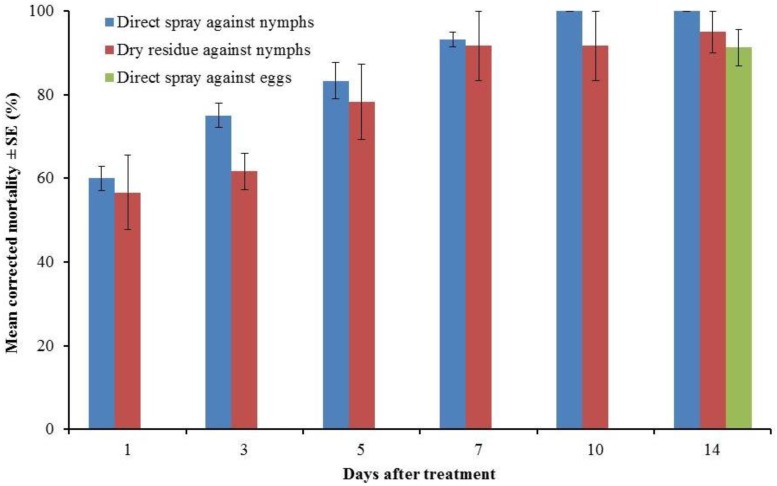
Efficacy of Alpine aerosol against Indy strain bed bugs in direct spray and dry residue exposure bioassays.

In the direct spray bioassay, Alpine aerosol caused 93.2 ± 1.7 and 100.0 ± 0.0% mortality in bed bug nymphs at 7 and 10 d after treatment, respectively (Figure 2). The mortality in the untreated control after 10 d was 3.3 ± 1.7%. Five-minute exposure to dry residue caused 91.6 ± 8.3, 91.6 ± 8.3, and 95.0 ± 5.0 % mortality to bed bug nymphs at 7, 10, and 14 d after treatment, respectively (Figure 2). No mortality occurred in the untreated control after 14 d. 

Direct spray of Alpine aerosol caused 91.3 ± 4.3% mortality of bed bug eggs (Figure 2). No egg hatching occurred after 7 d. Only 58.0 ± 30.0% of the hatched nymphs survived at 14 d after treatment. All eggs in the control group hatched and 98.7 ± 0.8% of the hatched nymphs survived at 14 d after treatment of the eggs. The bed bugs collected from the study apartments and the Indy strain bugs used in the bioassays had similar levels of resistance to deltamethrin in a separate experiment [19]. In that experiment, bed bugs were directly sprayed with 0.06% Suspend SC (a.i. deltamethrin) at 4.07 mg/cm^2^ under a Potter spray tower. The bed bugs collected from the study apartments and the Indy strain bed bugs suffered 29% and 35% mortality at 7 d after treatment, respectively, compared to 100% mortality in laboratory susceptible strain at 2 h after treatment. More than 90% mortality in Indy strain bed bugs by Alpine aerosol in the current study suggests the effectiveness of alpine aerosol in controlling pyrethroid resistant bed bugs. 

### 3.2. Effectiveness of a Reduced-Risk Insecticide based Bed Bug Management Program in Low-Income Housing

**Figure 3:**
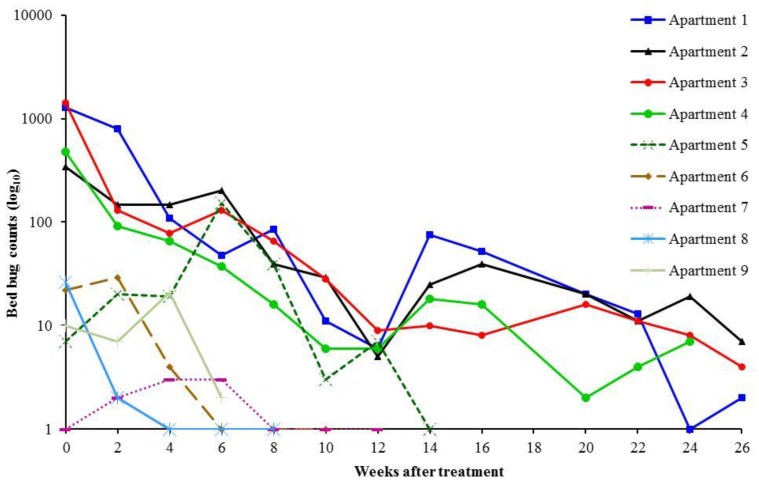
Bed bug count reduction (Climbup interceptors + visual inspection) in nine apartments after implementation of the bed bug management program.

**Figure 4:**
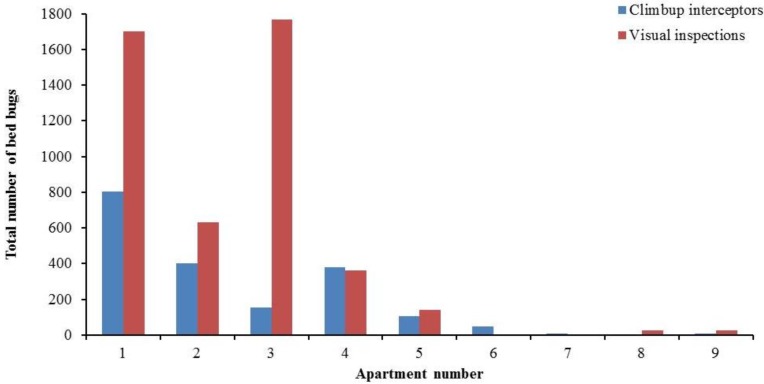
Total number of bed bugs counted from Climbup interceptors or visual inspections for each apartment during the entire study period.

Many units did not have bed frames to support the box springs and mattresses or the furniture legs were too large to install interceptors directly under the legs. In these units, interceptors + visual inspection provided more meaningful and accurate counts at each visit (Figure 3). However, total number of bed bugs found in interceptors or visual inspections during the entire study period for each apartment are also presented (Figure 4). Bed bugs found in the interceptors or visual inspections were both removed from the apartments or killed with steam or Alpine aerosol. 

The initial median (minimum, maximum) combined bed bug count based on interceptors and visual inspection of the nine apartments was 29 (1, 1,428). Four apartments were heavily infested (with ≥ 91 bed bugs). We spent a median (minimum, maximum) of 9 (4, 30) h to monitor and treat each infested apartment over the study period. A total of 283 g Alpine dust and 1,265 g Alpine aerosol were used in the nine apartments. Interceptors caught an average of 212.0 ± 90.2 bed bugs, whereas, visual inspections found 518.0 ± 240.4 bed bugs per apartment during the entire study (Figure 4). The IPM program was highly effective in reducing bed bug numbers. It resulted in an average of 96.8 ± 2.2% reduction in bed bug counts (Figure 3). However, elimination of bed bugs was only achieved in three apartments (Apartment 6–8) which had a low (1 to 29) number of bed bugs at the beginning (Table 1). The 9th apartment had low bed bug numbers at the beginning and still had two bed bugs at the end of the study. This unit was enrolled late and only received service for 6 wk. The four heavily infested apartments (Apartment 1–4) still had a few bed bugs even after 5.5–6.5 months’ service. Apartment 5 had only 20 bed bugs (interceptor count 17, visual count 3) during the initial inspection. However, the resident was recently moved in from a heavily infested unit when the unit was enrolled in this study. During one month post-treatment inspection, we found 125 bed bugs hiding in unpredicted areas such as book shelves, in cracks of tables, inside plastic totes, and on a picture frame. We suspect that the failure to eliminate bed bugs in this apartment was mainly due to the very scattered bed bug distribution resulting from the recent relocation of the resident’s infested belongings. 

**Table 1:** Effectiveness of a bed bug management program at low-income housing

Apartment no.	# occupants	# Climbups	Observation period (months)	Total treatment time (h)	Cooperation level *	Bed bug counts ^1^	
						Initial	Final
1	4	40	6.5	30	2	1,285	2
2	6	28	6.0	24	2	342	7
3	2	22	6.5	21	3	1,428	4
4	4	18	5.5	13	3	478	7
5	1	21	3.5	9	1	20	1
6	6	21	1.0	6	3	29	0
7	3	31	2.0	5	1	1	0
8	5	16	1.5	4	3	26	0
9	3	21	1.5	7	3	10	2

* Cooperation level: 1. very cooperative; 2. somewhat cooperative; 3. not cooperative; ^1^ Bed bug counts = Climbup interceptors + visual inspection.

The results achieved in this study are comparative to bed bug reduction and elimination rates reported in other IPM studies [3,7,8]. Wang *et al.* [3] reported bed bug reduction by 97.6 ± 1.6 and 89.7 ± 7.3% in the diatomaceous earth dust-based IPM and chlorfenapyr spray-based IPM, respectively. Bed bugs were eradicated from 50% of the apartments in each group after 10 wk. In a more recent study, the IPM program consisting of non-chemical treatment and 0.075% Temprid SC (imidacloprid and cyfluthrin), Tempo dust (1% cyfluthrin) or diatomaceous earth dust eliminated bed bugs from 44% of the apartments and reduced bed bug counts by 99.9% [7]. Wang *et al.* [8] evaluated another IPM program consisting of non-chemical treatment and chemical treatment (Tempo dust and Alpine aerosol). Bed bugs were eliminated from 25% of the apartments and bed bug counts were decreased by 92% over 12 wk. Our IPM program also resulted in a similar (96.8%) level of reduction compared to an insecticide only study reported by Potter *et al.* [6] (95.6%). Moreover, we achieved these results with a 96% reduction in pesticide usage compared to their study. Similarly, the current study reduced pesticide use by 94% compared to chemical only treatment in another study conducted in eight one-bedroom apartments in a high rise building [20].

All of the previously reported bed bug IPM studies [3,7,8] were conducted in studio or one-bedroom apartments with only one or two adults occupying each unit. What makes our study unique is that it was carried in family-style housing which is not only larger, but also presents a variety of different obstacles including, more beds, occupants, children and pets. Still we only used an average 172 g of formulated reduced risk-pesticide per apartment, much less than used in studies in smaller apartments but achieving similar levels of control.

Interception devices are typically thought of as a monitoring tool for the detection of bed bugs. However, in our study, interceptors caught an average of 43.7 ± 11.4% of the total bed bugs (interceptor + visual) found per apartment (Figure 4). In separate studies, Wang *et al.* [3,8] demonstrated the effectiveness of bed bug interceptors in reducing bed bug numbers. To what degree, interceptors contributed to overall population reduction is unclear and was not specifically measured in this study, however, we do believe that interceptors should be viewed not only as a detection tool but also as an important part of the IPM program from a control perspective. 

Pre-treatment interviews show 100% of the residents (n = 9) in the infested units were bothered by bed bug bites. The majority of residents described bed bug bites as itchy, painful, and caused loss of sleep. At the end of the project, nobody complained about bed bug bites despite the presence of low numbers of bed bugs in six apartments. All surveyed residents were satisfied with our service and level of control and hoped their apartments would continue to be inspected periodically for bed bugs. None of them used pesticides since we started this project. All residents indicated they learned some non-chemical methods for preventing or controlling bed bugs. 

The quality of the bed bug program was compromised by lack of cooperation by both the residents and property management. Only two out of nine residents were fully cooperative. For example, during each visit, bed bug infested bed linens and sheets were bagged and residents were asked to launder them. The majority of the residents did not launder and continued using infested sheets and linens from bags. Property management failed to provide encasements for all beds as initially agreed upon and did not provide access to all apartments during scheduled visits. These are some of the limitations associated with research in low-income housing, which makes the cooperative relationship required in a true IPM program challenging [7]. The results of this and previous studies suggest that while high level population reduction is possible with little cooperation from residents and management, the same is not true for successful elimination of bed bug infestations. Lack of cooperation contributes to slow elimination even with the best treatment efforts. Other challenges encountered in this study were: (1) presence of old furniture with many cracks, crevices, and holes; (2) lack of a bed frame to support the mattresses and box springs; and (3) relocating infested furniture within an apartment. The financial constraints, physical or mental challenges, and culture of the residents contributed to these conditions. No cooperation from residents in five out of nine apartments suggests our education did not significantly alter the residents’ level of cooperation during the study period (Table 1). Surprisingly, we did not find signs of bed bug infestations in the six apartments adjoining to the four heavily infested units after two consecutive biweekly inspections using interceptors and visual inspections. Wang *et al.* [4] found that 45% of the apartments in a high rise building became infested within 41 months of the first confirmed bed bug introduction and 53% of apartments adjacent to infested apartments had bed bugs. Among the infested apartments, an average of six bed bugs was detected dispersing through apartment entry doors every 4 wk. The lack of dispersal observed in our study might be due to the differences in building structure. There were only one or two adjacent units for each apartment in this study. 

Our study indicates infestations can be significantly reduced and sometimes eliminated without using large amounts of pesticides and that efficiency of elimination also appears to be associated with the extent of the infestation. Apartment (6–8) had low (1 to 29) number of bed bugs at the beginning. It took less than 2 months to eliminate bed bugs from these three units. In contrast, infestations in apartments with initial counts over 300 bed bugs (Apartment 1–4) were still not eliminated even after 5.5–6.5 months of service (Table 1). These four apartments had 16 to 86 bed bugs at two months after initial treatment (Figure 3). Although the sample size is small, the data clearly suggest the time required to eliminate an infestation is closely related to the infestation levels. Multiple visits and treatments are necessary to eliminate high level infestations. Similar findings were reported by Wang *et al.* [3] and Potter [15]. Therefore, proactive monitoring to identify unreported or new infestations is very important to prevent low level populations from becoming heavy infestations and to minimize the spread of bed bugs. 

## 4. Conclusions

Alpine aerosol direct spray and dry residue provided high level control of bed bugs in laboratory assays. Successful elimination of bed bug infestations is closely related to bed bug infestation levels, resident cooperation, and complexity of the apartments. An integrated pest management program incorporating targeted pesticide application will significantly reduce pesticide use and achieve high level of bed bug reductions. Periodical inspections using intercepting devices and visual inspections are important for evaluating program success and confirming elimination of bed bugs.
